# Smart Nanocomposites of Cu-Hemin Metal-Organic Frameworks for Electrochemical Glucose Biosensing

**DOI:** 10.1038/srep36637

**Published:** 2016-11-04

**Authors:** Juan He, Han Yang, Yayun Zhang, Jie Yu, Longfei Miao, Yonghai Song, Li Wang

**Affiliations:** 1 Key Laboratory of Functional Small Organic Molecule, Ministry of Education, College of Chemistry and Chemical Engineering, Jiangxi Normal University, 99 Ziyang Road, Nanchang 330022, People’s Republic of China

## Abstract

Herein, a smart porous material, Cu-hemin metal-organic-frameworks (Cu-hemin MOFs), was synthesized via assembling of Cu^2+^ with hemin to load glucose oxidase (GOD) for electrochemical glucose biosensing for the first time. The formation of the Cu-hemin MOFs was verified by scanning electron microscopy, X-ray powder diffraction, Fourier transform infrared spectroscopy, N_2_ adsorption/desorption isotherms, UV-vis absorption spectroscopy, fluorescence spectroscopy, thermal analysis and electrochemical techniques. The results indicated that the Cu-hemin MOFs showed a ball-flower-like hollow cage structure with a large specific surface area and a large number of mesopores. A large number of GOD molecules could be successfully loaded in the pores of Cu-hemin MOFs to keep their bioactivity just like in a solution. The GOD/Cu-hemin MOFs exhibited both good performance toward oxygen reduction reaction via Cu-hemin MOFs and catalytic oxidation of glucose via GOD, superior to other GOD/MOFs and GOD/nanomaterials. Accordingly, the performance of GOD/Cu-hemin MOFs-based electrochemical glucose sensor was enhanced greatly, showing a wide linear range from 9.10 μM to 36.0 mM and a low detection limit of 2.73 μM. Moreover, the sensor showed satisfactory results in detection of glucose in human serum. This work provides a practical design of new electrochemical sensing platform based on MOFs and biomolecules.

As an important research area of analytical chemistry^1^,^2^,^3^,^4^,^5^, electrochemical glucose biosensors have received significant attention over past few years because of their low cost, quick response, simple preparation and wide applications in biomedical, clinical research, food production, ecology and even textile industry^6^,^7^,^8^,^9^. Particularly, glucose oxidase (GOD)-based glucose biosensors have been one of the hot spots in analytical chemistry as the introduction of nanomaterials^10^,^11^.

Various nanomaterials (such as graphene^12^, carbon nanotubes^13^, metal or metal oxide nanostructures^14^ as well as their nanocomposites) have been extensively employed to immobilize GOD on electrode surface for developing electrochemical glucose biosensors owing to their high specific surface area, fast electron transfer, good affinity for enzymes/proteins, excellent catalytic activity^15^ and remarkable biocompatibility^7^,^16^,^17^. As previously reported, nanomaterials could both improve the direct electron transfer between the eletroactive center of enzymes/proteins and the electrode surface, and load enzymes/proteins on the electrode surface effectively^18^,^19^,^20^,^21^,^22^. However, nanomaterials always form close-packed structures after they are assembled on electrode surface, which hinders their performance greatly^23^,^24^,^25^,^26^,^27^,^28^. Furthermore, most of GOD-based biosensors detect glucose level via monitoring the consumption of O_2_ in enzymatic reaction^29^. So, the nanomaterials are used not only as a supporting to load GOD molecules but also as a catalyst for the reduction of O_2_ in which the amount of O_2_ consumed in the enzymatic reaction could be explored^19^,^30^,^31^. Paradoxically, excess GOD immobilized on nanomaterials could enhance the performance of GOD, yet hinder the reduction of O_2_, which still results in a poor performance. Thus, there are still challenges to develop new materials for constructing GOD-based glucose biosensors.

Metal-organic frameworks (MOFs) and zeolitic imidazolate frameworks (ZIFs) own perfect physical and chemical properties such as three-dimensional (3D) structure, high specific surface area, multiple inner porosity and high crystalline. They have been extensively applied in gas storage and separation, drug delivery, catalysis, clinical diagnosis, chemical sensors and analysis^17^,^32^,^33^,^34^,^35^,^36^,^37^. Recently, the MOFs/ZIFs have been used to load enzymes/proteins into their pores for enhancing the activity, recyclability and solvent adaptability of enzymes/proteins^38^. A series of MOFs with differently sized pores were also synthesized via changing the chain length of ligands for loading various biomolecules^39^,^40^,^41^,^42^. Some biomolecule@MOFs/ZIFs have been applied for biosensing. For example, Liu Y. *et al.* synthesized hemin@MIL–101(Al)–NH_2_ to combine with GOD for colorimetric detection of glucose^43^. Liu Z. *et al.* has also prepared cytochrome c/ZIF-8 by one–pot synthesis for glucose and H_2_O_2_ detection by colorimetric method^42^. The previous results clearly indicated that small molecules and electrolyte could still enter into the pores of MOFs/ZIFs freely after the biomolecules was loaded into their pores. However, only a few enzymes are loaded into the MOFs/ZIFs, which results in a narrow linear range. These studies activate us to construct an electrochemical glucose biosensor by using MOFs as immobilizing matrix to load GOD.

Herein, a novel Cu-hemin MOFs with an excellent catalytic activity toward the reduction of O_2_ was fabricated via Cu^2+^ coordinating with hemin for electrochemical glucose biosensing. The novel Cu-hemin MOFs showed a 3D ball-flower-like nanostructure and was hollow inside, which could be used to load a large number of GOD molecules. The GOD molecules were incorporated into Cu-hemin MOFs by utilizing the associated pores of MOFs, which effectively avoided the aggregation of enzyme on the surface of electrode. The ball-flower-like nanostructure of Cu-hemin MOFs resulted in the contact of GOD molecules with glucose and electrolyte to keep the catalytic activity of enzyme. The GOD/Cu-hemin MOF nanocomposites could not only catalyze the reduction of O_2_ via the Cu-hemin MOFs effectively but also catalyze the oxidization of glucose via GOD, avoiding the shortcomings of traditional nanomaterials and other MOFs/ZIFs. The as-prepared glucose sensor based on the GOD/Cu-hemin MOFs exhibited wide linear range, low detection limit, excellent sensitivity and good selectivity.

## Results and Discussion

**Figure 1:**
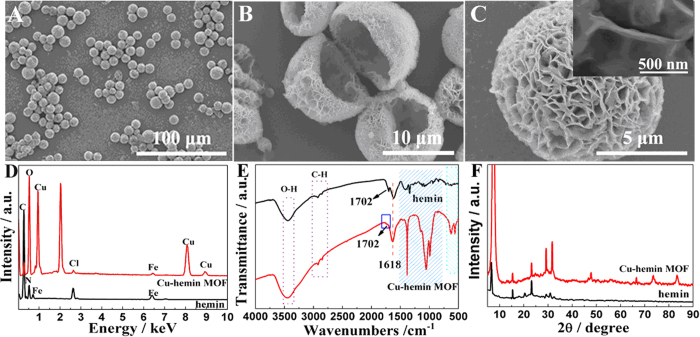
SEM images of Cu-hemin MOFs prepared at the mole ratio of Cu^2+^ and hemin of 171:1 at (**A**) Low- and (**B**,**C**) high-magnification. Insert in Fig. 1C showed detailed structure. (**D**) EDXS, (**E**) FT-IR spectra and (**F**) XRD patterns of hemin and Cu-hemin MOFs.

As shown in Fig. 1A, a large number of uniform 3D Cu-hemin MOFs with ball-flower-like structure appeared. From the low magnification scanning electron microscopy (SEM) image (Fig. 1A), it could be seen that the diameter of the 3D flower-like ball was about 10 μm. Meanwhile, it could also be seen that the Cu-hemin MOFs showed a big hollow cage and a large number of holes in the flower-like ball, which could not only provide a large specific surface area but also enhance the mass transfer (Fig. 1B). Moreover, the details of the fold structure could be distinctly revealed by the high magnification SEM image (Fig. 1C) and the average thickness of these flakes was about 50 nm (insert of Fig. 1C). Due to different linkages between metal ions and organic ligands, MOFs could present various morphologies including rod octahedron^42^,^44^, nanosphere^45^, hexahedron^45^,^46^, spindle-shape^47^, and so on. However, such ball-flower-like structure has not yet been reported in previous works. The special structure of hemin might result in the formation of such ball-flower-like structure via a linkage between Cu^2+^ and carboxy groups of hemin. As a fixed-structure molecule, hemin owned three feature elements (Fe, N and Cl) which could be used to prove its existence^36^,^48^,^49^. As could be seen from energy dispersive X-ray spectroscopy in Fig. 1D, the apparent peaks of Fe, N, Cl and Cu were observed in the solid products, which declared the formation of Cu-hemin MOFs. In order to verify this conclusion, Fourier transform infrared spectroscopy (FT-IR) and the X–ray powder diffraction (XRD) test were carried out. In the FT-IR spectra (Fig. 1E), both hemin and Cu-hemin MOFs exhibited obvious peaks for O-H at 3442 cm^−1^. The band at 1618 cm^−1^ originated from C=C and C=N of the protoporphyrin (IX) ring system^25^. The weak peak at 1701 cm^−1^ was ascribed to C=O stretching vibration, which was smaller in the curve of Cu-hemin MOFs due to the coordination between −COOH groups and Cu^2+^. The strong peak at 1384 cm^−1^ belonged to −CH_3_ of hemin was stronger in the curve of Cu-hemin MOFs due to the ordered assembly of hemin in the Cu-hemin MOFs^50^. Hence, FT-IR spectra also proved the formation of Cu-hemin MOFs. The XRD spectra of hemin and Cu-hemin MOFs were exhibited in Fig. 1F 51,52. Some diffraction peaks appeared in the pattern of Cu-hemin MOFs, suggesting that the 3D-flower-like ball nanostructures was crystalline materials and the crystal structure of Cu-hemin MOFs was similar to that of previous hemin-MOFs^37^. The XPS of Cu-hemin MOFs exhibited in Figure S1 (Supporting Information) has also proved the successful synthesis of Cu-hemin MOFs. The detailed discussion on the crystal structure of Cu-hemin MOFs will be reserved in the future study. All the results confirmed that the 3D-flower-like ball nanostructures was the Cu-hemin MOFs which had an ordered structure and high crystalline.

**Figure 2:**
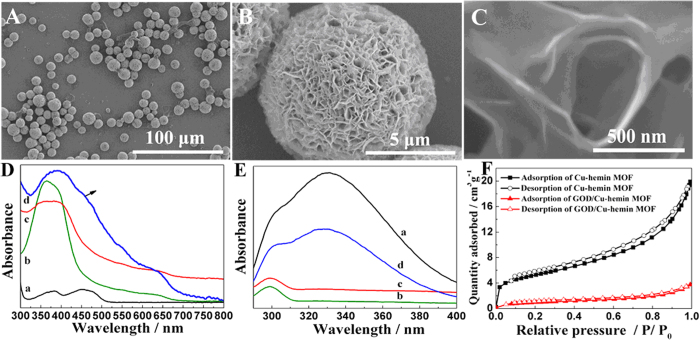
SEM images of GOD/Cu-hemin MOFs nanocomposites at (**A**) Low- and (**B**,**C**) high-magnification. (**D**) UV-vis spectra and (**E**) Fluorescence spectra of GOD (**a**), hemin (**b**), Cu-hemin MOFs (**c**) and GOD/Cu-hemin MOFs nanocomposites (**d**). (**F**) N_2_ adsorption/desorption isotherms measured at 77 K for the Cu-hemin MOFs (black curve) and GOD/Cu-hemin MOFs nanocomposites (red curve).

The SEM images of GOD/Cu-hemin MOFs nanocomposites showed a similar diameter and morphology to that of Cu-hemin MOFs (Fig. 2A). Magnified SEM images further confirmed that the Cu-hemin MOFs maintained its framework integrity after loading GOD molecules in the pores (Fig. 2B,C), which was also confirmed by the XRD studies (Figure S2A, Supporting Information). The UV-vis absorbance spectrum of hemin (curve b, Fig. 2D) revealed a strong absorption peak at 367 nm of Soret band and a weak absorption peak at about 652 nm of Q-band^25^,^52^. Two absorption peaks also appeared at the spectrum of Cu-hemin MOFs (curve c) and GOD/Cu-hemin MOFs nanocomposites (curve d), indicating the structure of hemin was maintained. A doublet peaks at 375 nm and 455 nm appeared in the spectrum of GOD (curve a)^7^,^53^. For GOD/Cu-hemin MOFs nanocomposites, a weak peak at 457 nm was observed and the peak at 375 nm might be covered by the strong peak at 367 nm, which indicated that GOD was successfully loaded into Cu-hemin MOFs. No obvious shift of peaks in the GOD/Cu-hemin MOFs nanocomposites was observed (curve d), which might indicate the structure of GOD was well kept. Since the carboxyl groups of hemin were coordinated with Cu^2+^ to form Cu-hemin MOFs, the amine of GOD could not link with carboxyl groups of hemin and accordingly GOD molecules might be loaded in the pores of Cu-hemin MOFs. Figure 2E displayed a steady-state fluorescence spectra of these materials, and it revealed that only GOD (curve a) and GOD/Cu-hemin MOFs nanocomposites (curve d) showed strong emission peak at 339 nm. The decrease of density in fluorescence spectrum of GOD/Cu-hemin MOFs nanocomposites might indicate that GOD molecules were loaded in the pores of Cu-hemin MOFs. N_2_ adsorption/desorption isotherms (Fig. 2F) test under 77 K were carried out and showed that the Brunauer-Emmett-Teller (BET) surface area of Cu-hemin MOFs decreased from 17.00 to 3.08 m^2 ^g^−1^ after the loading of GOD. The result further confirmed that GOD molecules were loaded in the pores of Cu-hemin MOFs. The FT-IR spectra and thermogravimetric data (TGA) curves (Figure S2B,C, Supporting information) also confirmed the conclusion.

**Figure 3:**
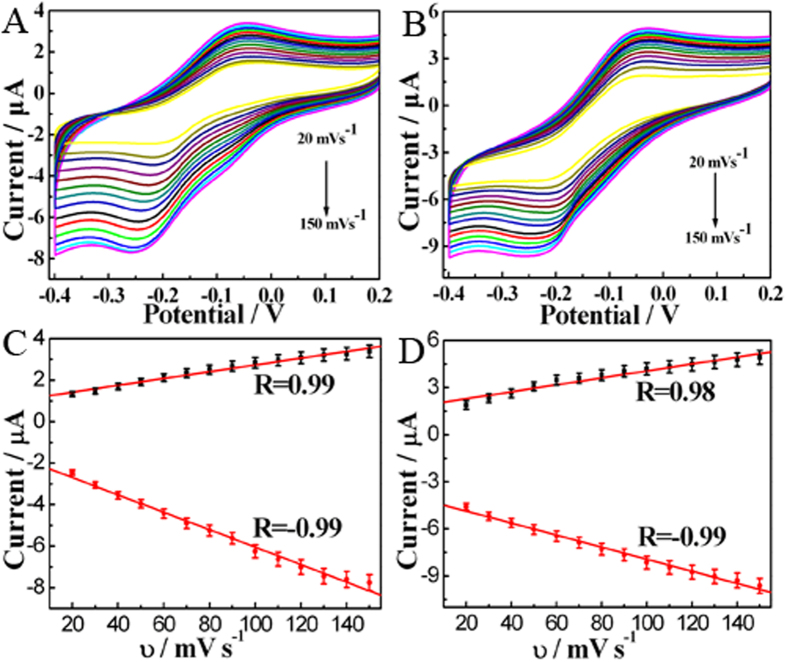
CVs of (**A**) Cu-hemin MOFs/GCE and (**B**) GOD/Cu-hemin MOFs/GCE in 0.1 M N_2_-saturated PBS (pH = 7.0) at different scan rates by step of 10 mV s^−1^. Plot of peak current versus the scan rates for (**C**) Cu-hemin MOFs/GCE and (**D**) GOD/Cu-hemin MOFs nanocomposites/GCE.

Electrochemical properties of Cu-hemin MOFs/glassy carbon electrode (GCE) and GOD/Cu-hemin MOFs nanocomposites/GCE at different scan rates were investigated by cyclic voltammograms (CVs) which both showed a pair of distinct redox peak of hemin (Fig. 3A,B)^15^,^36^,^48^,^54^. The peak currents (*I*_p_) displayed good linear correlations with scan rates (*υ*) from 20 to 150 mV s^−1^, suggesting quasi-reversible surface-controlled processes (Fig. 3C,D). The results clearly indicated that electrochemical properties of hemin were not changed in both Cu-hemin MOFs and GOD/Cu-hemin MOFs nanocomposites. It could be ascribed to the porous structure of Cu-hemin MOFs and GOD/Cu-hemin MOFs nanocomposites which enhanced the mass transfer effectively. After GOD was loaded in Cu-hemin MOFs, the peak current decreased slightly, indicating GOD molecules was successfully loaded in the pores of Cu-hemin MOFs. In the potential range, the typical redox peaks of GOD molecules were not observed. It is well known that the redox center of GOD is obstructed by the protein shell and the electron transport rate between the active site of GOD and electrode surface is slow. The direct electron transfer could be achieved by immobilizing GOD molecules on nanomaterials where the secondary structure of GOD molecules was changed to reveal the eletroactive center but their bioactivity was lost^19^. Thus, the missing of redox peaks for GOD molecules might indicate their bioactivity was well kept in the GOD/Cu-hemin MOFs nanocomposites.

Electrochemical impedance spectroscopy (EIS) was used to explore the electron transfer of various electrodes and the results were shown in Figure S3A (Supporting Information). The Randles circuit (Inset of Figure S3A, Supporting Information) was used for matching with the impedance records. The resistance of charge transfer (*R*_*ct*_) of the bare GCE (147 Ω, curve a) as indicated by the Nyquist circle on the curve was very small. The *R*_*ct*_ of Cu-hemin MOFs/GCE (3079 Ω, curve b) increased greatly due to its poor conductivity. After the loading of GOD, the *R*_*ct*_ was further increased to 7094 Ω (curve c) which might be attributed to the blocking effects of negatively charged biomacromolecule of GOD on Fe(CN)_6_^3−/4−^. The result also confirmed that GOD molecules were successfully loaded in the pores of Cu-hemin MOFs.

**Figure 4:**
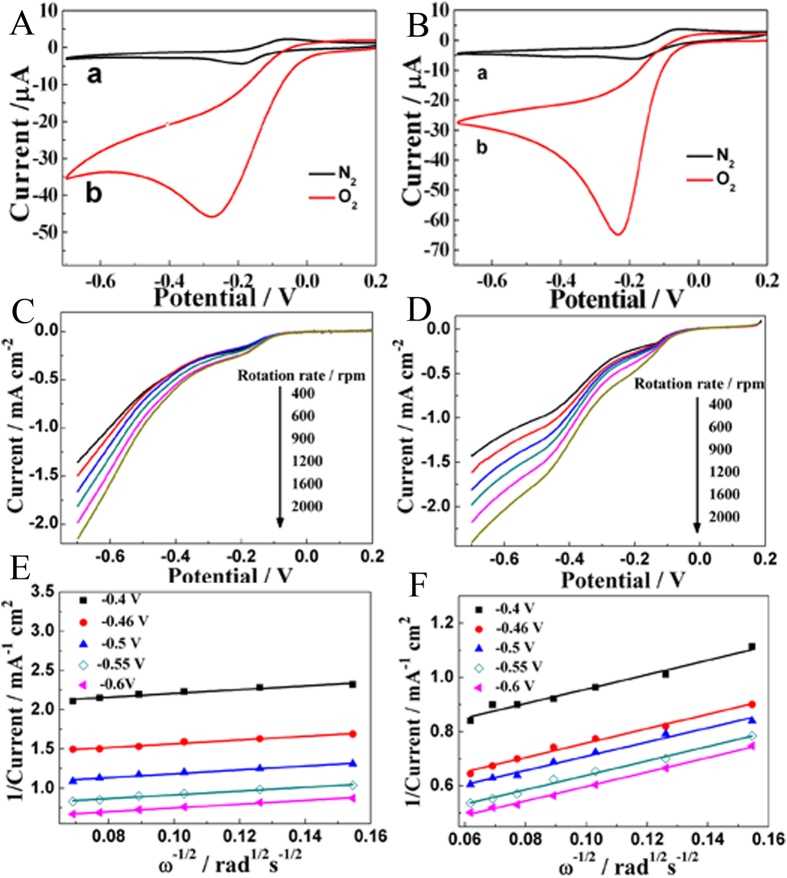
CVs of (**A**) Cu-hemin MOFs/GCE and (**B**) GOD/Cu-hemin MOFs/GCE in 0.1 M N_2_-saturated (black curve) and O_2_-saturated (red curve) PBS (pH = 7.0). Scan rate: 50 mV s^−1^. RDE LSV of (**C**) Cu-hemin MOFs/GCE and (**D**) GOD/Cu-hemin MOFs/GCE in 0.1 M O_2_-saturated PBS (pH = 7.0) with various rotation rates. Koutecky-Levich plots at different electrode potentials for (**E**) Cu-hemin MOFs/GCE and (**F**) GOD/Cu-hemin MOFs/GCE.

Since most of enzymatic glucose biosensors are used to detect glucose level via monitoring the consumption of O_2_ in enzymatic reaction, it is very important to explore the electrocatalytic activity of GOD/Cu-hemin MOFs nanocomposites and Cu-hemin MOFs toward ORR. Figure 4A,B showed CVs of Cu-hemin MOFs/GCE and GOD/Cu-hemin MOFs nanocomposites/GCE in 0.1 M N_2_/O_2_-saturated PBS at a scan rate of 50 mV s^−1^ for oxygen reduction reaction (OOR), respectively. As shown in Fig. 4A, the anodic peak was diminishing with the increased O_2_ in electrolytes and the reductive peak became obvious with a slight positive shift, showing a typical ORR. After GOD molecules were loaded on the Cu-hemin MOFs, the reductive peak current became larger and the peak potential positively shifted as compared with that of the Cu-hemin MOFs (Fig. 4B), indicating GOD might be also involved in the catalytic process. The reductive peak current of GOD/Cu-hemin MOFs nanocomposites/GCE was also larger than that of hemin/GCE where the same amount of hemin was modified on the electrode surface (Figure S3B, Supporting Information), suggesting the electrocatalytic performance toward O_2_ was greatly enhanced when hemin was used to construct the Cu-hemin MOFs. The large peak current could be used as significantly amplified signal for glucose detection. The effect of GOD amount on the electrocatalytic performance toward O_2_ was studied by the cathodic peak on CVs (Figure S4A, Supporting Information), which indicated the maximum cathodic peak was at 1.5 nmol GOD. In this work, the influence of pH was also explored from 6.0 to 8.0 (Figure S4B, Supporting Information), which displayed a optimum electrocatalytic current at pH 7.0. It could be ascribed to the fact that the optimal pH of the enzymatic reaction was pH 7.0 (that is, the bioactivity of GOD was largest at pH 7.0).

To obtain the electron transfer number of ORR by Cu-hemin MOFs and GOD/Cu-hemin MOFs nanocomposites, reaction kinetics were investigated by rotating-disk electrode (RDE) linear sweep voltammograms (LSV). The current density was enhanced by increasing of rotation rate from 400 to 2000 rpm (Fig. 4C,D). The corresponding Koutecky− Levich plots (*J*^−*1*^ vs *ω*^−*1/2*^) under different electrode potentials showed a good linearity (Fig. 4E,F). Linearity and parallelism of the plots were considered as typical of first-order reaction kinetics with respect to the dissolved O_2_. The kinetic parameters could be calculated based on the Koutecky−Levich equations:^25^


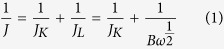



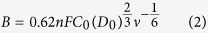



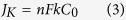


in which *J* is the measured current density, *Ј*_*K*_ and *Ј*_*L*_ are the kinetic and diffusion-limiting current densities, *ω* is the angular velocity of the disk (*ω* = 2*π*N, N is the linear rotation speed), *n* is the overall number of electrons transferred in oxygen reduction, *F* is the Faraday constant (*F* = 96485 C mol^−1^), *C*_*0*_ is the bulk concentration of O_2_, *ν* is the kinematic viscosity of the electrolyte, *D*_*0*_ is the coefficient of diffusion for O_2_, and the *k* is the electron transfer rate constant. *n* and *Ј*_*K*_ could be obtained from the slope and intercept of the Koutecky− Levich plots in Fig. 4E,F (parameters *C*_*0*_ = 1.2 × 10^−3 ^mol L^−1^, *D*_*0*_ = 1.9 × 10^−5 ^cm s^−1^, and *υ* = 0.01 cm^2 ^s^−1^ in 0.1 M PBS), respectively. Then *n* was estimated to be 3.78 and 3.41 for Cu-hemin MOFs and GOD/Cu-hemin MOFs nanocomposites, respectively. The result indicated that a direct 4-electron reduction pathway was obtained for ORR.

**Figure 5:**
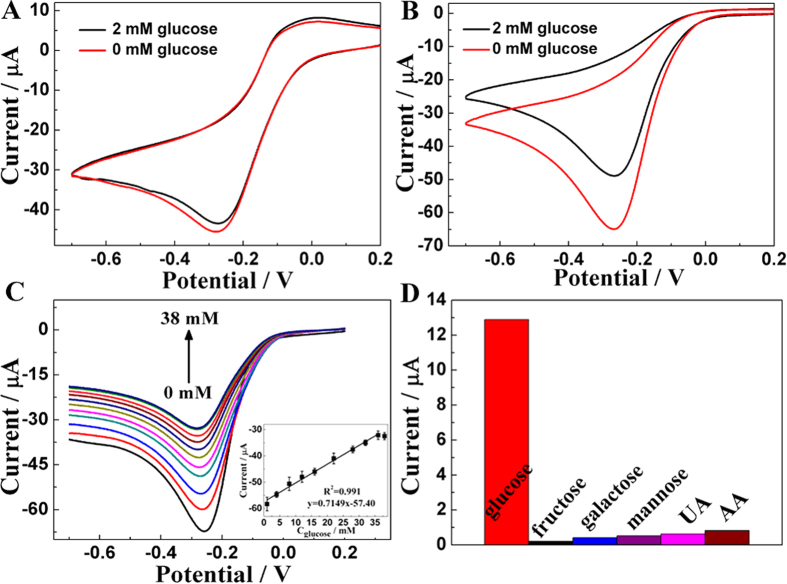
(**A**) Cu-hemin MOFs/GCE and (**B**) GOD/Cu-hemin MOFs/GCE in 0.1 M O_2_-saturated PBS (pH = 7.0) in the absence (red curve) and presence (black curve) of 2 mM glucose. (**C**) LSV of GOD/Cu-hemin MOFs/GCE in 0.1 M O_2_-saturated PBS (pH = 7.0) in the presence of glucose with various concentrations. Insert: Plot of peak current versus the concentration of glucose. (**D**) Comparison of the change of CVs response of GOD/Cu-hemin MOFs/GCE in 0.1 M O_2_-saturated PBS (pH = 7.0) containing 0.2 mM glucose and 2 mM various interferences. Scan rate: 50 mV s^−1^.

In order to further discuss whether GOD/Cu-hemin MOFs nanocomposites/GCE could catalyze glucose, a study was carried out in the presence and absence of 2 mM glucose in 0.1 M O_2_-saturated PBS (Fig. 5A,B). It displayed an obvious decrease of cathodic current at the GOD/Cu-hemin MOFs nanocomposites/GCE (Fig. 5B) while only a slight decrease of cathodic current occurred at the Cu-hemin MOFs/GCE (Fig. 5A), exhibiting a typical electrocatalysis of glucose by GOD. The good catalytic performance might result from both a large number of GOD loaded in the Cu-hemin MOFs effectively and the good bioactivity of GOD remained in Cu-hemin MOFs. In Figure S5A,B (Supporting Information), CVs was carried out to further explore the bioactivity of GOD, where the current change was similar to free GOD and GOD/Cu-hemin MOF nanocomposite after glucose was added into the electrolyte solution. Meanwhile, a wide linear range of GOD/Cu-hemin MOFs/GCE from 9.10 μM to 36.0 mM (*R* = 0.99, S/N = 3) was achieved (Fig. 5C). And the detection limit was estimated to be 2.73 μM with a higher sensitivity of about 22.77 μA mM^−1 ^cm^−2^. All these results exhibited superiority to other GOD-based glucose sensor as compared in Supplementary Table S1 (Supporting Information).

Figure 5D showed chemicals including fructose, galactose, mannose, uric acid (UA) and ascorbic acid (AA) in ten times concentration of glucose did not interfere with glucose detection. The result indicated that the GOD/Cu-hemin MOFs nanocomposite/GCE had a perfect selectivity for glucose detection. As shown in Table S2 (Supporting Information), the detection of glucose in human serum sample was studied by adding glucose into human serum solution which was diluted by 0.1 M PBS. The results of recovery indicated that the GOD/Cu-hemin MOFs nanocomposite/GCE was reliable and sensitive enough for real sample detection. The concentration of glucose in human serum sample of diabetes mellitus patients has also been evaluated to be about 8.32 mM, close to the value obtained from the clinical results with a relative deviation of 0.43%.

The long-term stability of GOD/Cu-hemin MOFs nanocomposite/GCE was explored, and the biosensor retained 99.6% of original current after the biosensor was stored at 4 °C for 48 h and 87.5% of original current for 30 days (Figure S6, Supporting Information). The reproducibility of the biosensors was also evaluated from the response toward 2 mM glucose at with five electrodes and the relative standard deviation (RSD) was about 4.27%. The results indicated that the biosensor showed a good long-term stability and reproducibility for glucose detection.

In summary, a novel ball-flower-like Cu-hemin MOFs with excellent catalytic activity toward the reduction of O_2_ was fabricated via the coordination of Cu^2+^ with hemin for the first time. The porous Cu-hemin MOFs could be used as supporting materials to load GOD effectively for fabricating glucose biosensor. The porous hollow structure of GOD/Cu-hemin MOFs nanocomposites enhanced the mass transfer and improved the efficiency of active GOD molecules, and the bioactivity of GOD could also be kept in the GOD/Cu-hemin MOFs nanocomposites. The GOD/Cu-hemin MOFs nanocomposites could not only catalyze the reduction of O_2_ via the Cu-hemin MOFs but also catalyze the oxidization of glucose via GOD, which successfully avoided the drawback of nanomaterials as supporting to load GOD. As a consequence, the as-prepared glucose sensor based on the GOD/Cu-hemin MOFs nanocomposites exhibited wide linear range, low detection limit, excellent sensitivity and good selectivity. Overall, the proposed method to prepare Cu-hemin MOFs nanocomposites is simple, efficient and easy to mass production. It might open up a new way for glucose sensors and shed new light on MOFs-based biosensors.

## Methods

### Materials

Hemin and copper nitrate trihydrate (Cu(NO_3_)_2_·3H_2_O) were purchased from Aladdin Reagent Co., Ltd (Shanghai, China). Glucose oxidase (GOD, EC 1.1.3.4, 140 U mg^−1^) and human serum were purchased from Sigma-Aldrich. The other chemicals were obtained from Beijing Chemical Reagent Factory (Beijing, China) and were of analytical grade without further purification. Phosphate buffer solution (PBS, 0.1 M) was prepared by mixing 0.2 M Na_2_HPO_4_ and 0.2 M NaH_2_PO_4_. Hemin solution (0.50 mM) was obtained by ultrasonic dissolving hemin in 0.1 M NaOH then the pH was adjusted to 7.0. GOD solutions (62.5 μM) were obtained by dissolving GOD in 0.1 M PBS (pH 7.0). The solution of hemin and GOD were stored in refrigerator before use. Ultra-pure water was purified by a Millipore-Q System (ρ ≥ 18.2 MΩ cm^−1^) and used in the whole experiments.

### Instrumentation

SEM and EDXS characterizations were operated on a HITACHI S-3400N scanning electron microscope with a Phoenix energy X-ray analyzer. XRD data were collected on a D/Max 2500 V/PC X–ray powder diffractometer using Cu Kα radiation (*λ* = 0.154056 nm, 40 kV, 200 mA). FT-IR spectroscopy was obtained by a Perkin-Elmer Spectrome 100 spectrometer (Perkin-Elmer Company, USA) with KBr power. N_2_ adsorption/desorption isotherms were measured at 77 K in a liquid nitrogen atmosphere using a Tristar 3000 volumetric adsorption analyzer (Micromeritics Instrument Corporation, USA) after the samples were pretreated at 200 °C for 12 h under vacuum. TGA were conducted on SDT 2960 with a heating rate of 10 °C min^−1^ under N_2_. UV-vis absorption spectra were recorded on a Hitachi U-3900H UV-vis Spectrophotometer. Fluorescence spectroscopy was determined on a Fluorescence Spectrophotometer (Hitachi) F-7000.

All electrochemical measurements were performed on a CHI 660C electrochemical workstation (Shanghai, China) at ambient temperature. A common three electrode system was employed including a bare or modified GCE as the working electrode, a platinum wire as the auxiliary electrode and a saturated calomel electrode (SCE) as the reference electrode. CVs and LSV were performed in a quiescent 0.1 M PBS (pH 7.0). EIS results were operated in 5 mM Fe(CN)_6_^3−/4−^ solution including 0.1 M KCl as the supporting electrolyte. Rotating disk electrode (RDE) measurements were performed in O_2_-saturated 0.1 M PBS (pH 7.0) solution with rotation rates from 400 to 2000 rpm at a scan rate of 10 mV s^−1^ on a GC disk electrode (Pine Instruments, 5.0 mm in diameter, A = 0.2 cm^2^) as working electrode, a Pt plate as the counter electrode and a Ag/AgCl (saturated KCl) as the reference electrode.

### Preparation of of GOD/Cu-hemin MOFs Nanocomposites

0.04 M Cu(NO_3_)_2_·3H_2_O solution and 0.50 mM hemin solution were mixed together with a volume ratio of 2:1 at room temperature and reacted for about 2 h. The produced ash green powder was acquired by centrifugation, washed with water and dried at 50 °C overnight. Then 24 μL 62.5 μM GOD was added into 150 μL suspension of Cu-hemin MOFs for 24 h at 4 °C. The resulted GOD/Cu-hemin MOFs powder was acquired by centrifugation, washed with 0.1 M PBS (pH 7.0) and dried at room temperature for overnight. The conditions to prepare Cu-hemin MOFs and GOD/Cu-hemin MOFs was optimized and the results were shown in Figure S7 (Supporting Information). As shown in Figure S7, as the ratio of Cu^2+^ and hemin was increased from 10:1 to 171:1 (Figure S7A–C, Supporting Information), the nanostructure became more and more uniform and finally some uniform ball-flower-like nanostructures appeared. After the mole ratio of Cu^2+^ and hemin was increased to 513:1 (Figure S7D, Supporting information), some nanostructures were damaged. Although some small Cu-hemin MOFs could be observed at 10:1, the Cu-hemin MOFs was not uniform and some deformed Cu-hemin MOFs were also observed, which was bad for the electrochemical performance. Therefore, the Cu-hemin MOFs prepared at the mole ratio of Cu^2+^ and hemin of 171:1 was used to construct the electrochemical biosensors.

### Preparation of the Modified Electrode

The bare GCE (3 mm in diameter) was polished with 1.0 and 0.3 μm alumina powder, ultrasonic washed in pure water for 5 min, then dried under a high-purity N_2_ stream. The modified GCE were prepared by dropping 9 μL Cu-hemin MOFs or GOD/Cu-hemin MOFs aqueous suspension on the surface of the polished GCE and then dried at 4 °C. Next, 1.0 μL of 0.05% nafion solution were dropped onto the modified GCE surface and subsequently dried at 4 °C for 4 h. Finally, the modified electrode was immersed into 0.1 M PBS (pH 7.0) to remove those weakly bound molecules and obtain the nanocomposites modified target electrode.

## Additional Information

**How to cite this article**: He, J. *et al.* Smart Nanocomposites of Cu-Hemin Metal-Organic Frameworks for Electrochemical Glucose Biosensing. *Sci. Rep.* **6**, 36637; doi: 10.1038/srep36637 (2016).

**Publisher’s note:** Springer Nature remains neutral with regard to jurisdictional claims in published maps and institutional affiliations.

## Supplementary Material

Supplementary Information
