# Supplementary material for: Smart Nanocomposites of Cu-Hemin Metal-Organic Frameworks for Electrochemical Glucose Biosensing
Source: Sci Rep. 2016 Nov 4;6:36637. doi: 10.1038/srep36637 (PMC5095656; doi:10.1038/srep36637)
Supplement: Supplementary Information [file srep36637-s1.doc]

Supporting Information

**Smart Nanocomposites of Cu-Hemin Metal-Organic Frameworks for Electrochemical Glucose Biosensing**

Juan He, Han Yang, Yayun Zhang, Jie Yu, Longfei Miao, Yonghai Song* and Li Wang[[1]](#footnote-2)*

*Key Laboratory of Functional Small Organic Molecule, Ministry of Education, Key Laboratory of Chemical Biology, Jiangxi Province, College of Chemistry and Chemical Engineering, Jiangxi Normal University, 99 Ziyang Road, Nanchang 330022, China.*

**Contents**

XPS of Cu-hemin MOFs……………………………………………….…………………**Figure S1**

XRD, FT-IR and TGA of Cu-hemin MOFs and GOD/Cu-hemin MOFs…………………**Figure S2**

EIS and CVs of different electrodes………………………………………………………**Figure S3**

Effects of the molar of GOD and pH.……………………………………………………**Figure S4**

LSV of Cu-hemin MOF/GCE and GOD/Cu-hemin MOF/GCE……………………………**Figure S5**

**The stability of GOD/Cu-hemin MOF/GCE**……………….……………………………**Figure S6**

SEM images of Cu-hemin MOFs and GOD/Cu-hemin MOFs……………………………**Figure S7**

Comparison of the performance of various GOD-based glucose sensors…………………**Table S1**

Determination of glucose in blood serum sample……………………………………………**Table S2**

Determination of glucose in blood serum sample of diabetes mellitus patients…………….**Table S3**


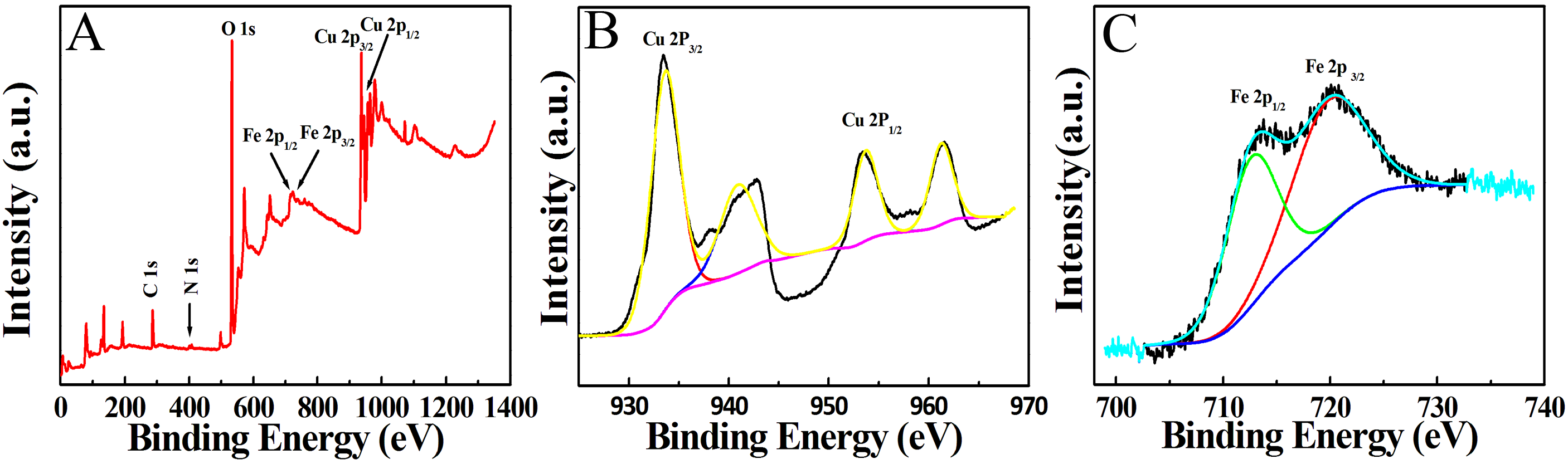


**Figure S1**. The XPS spectra of Cu-hemin MOF (A) and the high-resolution XPS spectrum for Cu (B) and Fe (C).


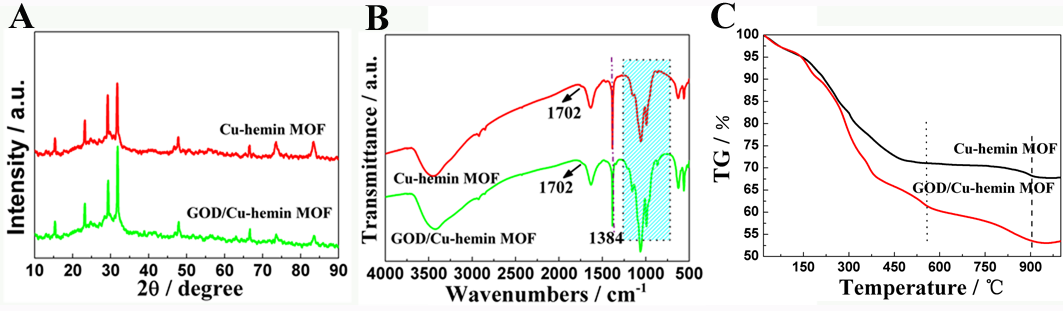


**Figure S2**. (A) XRD patterns, (B) FT-IR spectra and (C) TGA at nitrogen atmosphere of Cu-hemin MOFs and GOD/Cu-hemin MOFs.


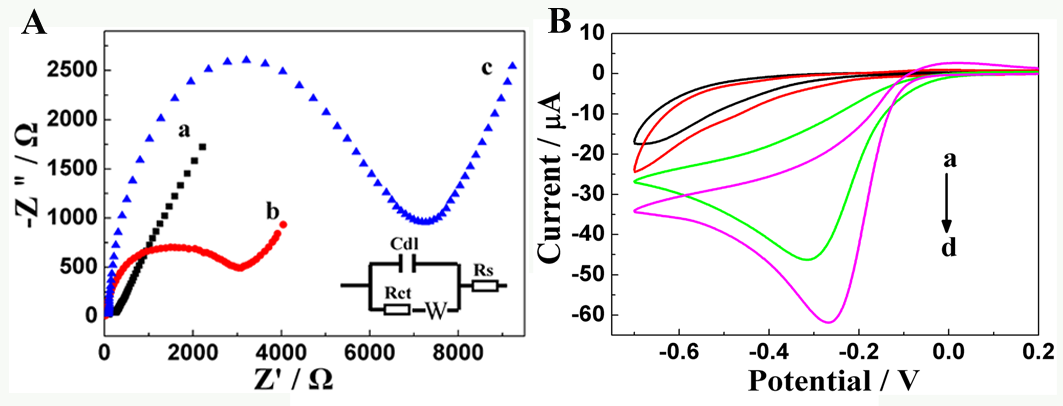


**Figure S3.** (A) EIS of (a) bare GCE, (b) Cu-hemin MOFs and(c) GOD/Cu-hemin MOFs in 0.1 M KCl solution containing 5.0 mM Fe(CN)_6_^3-/4-^. (B) CVs of bare (a) GCE, (b) GOD/GCE, (c) hemin/GCE and (d) GOD/Cu-hemin MOFs/GCE in 0.1 M O_2_-saturated PBS (pH=7.0) solution. Scan rate: 50 mV s^-1^.


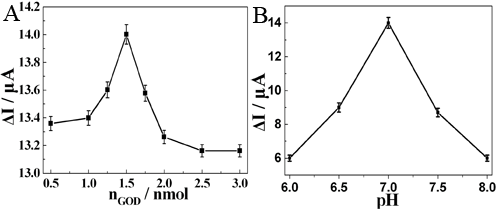


**Figure S4**. (A) Effects of GOD amount on the catalytic current of GOD/Cu-hemin MOFs/GCE in 0.1 M O_2_-saturated PBS (pH=7.0) with 2 mM glucose. (B) Plot of peak current versus different pH in 0.1 M O_2_-saturated PBS in the presence of 2 mM glucose for GOD/Cu-hemin MOFs/GCE. Scan rate: 50 mV s^-1^.


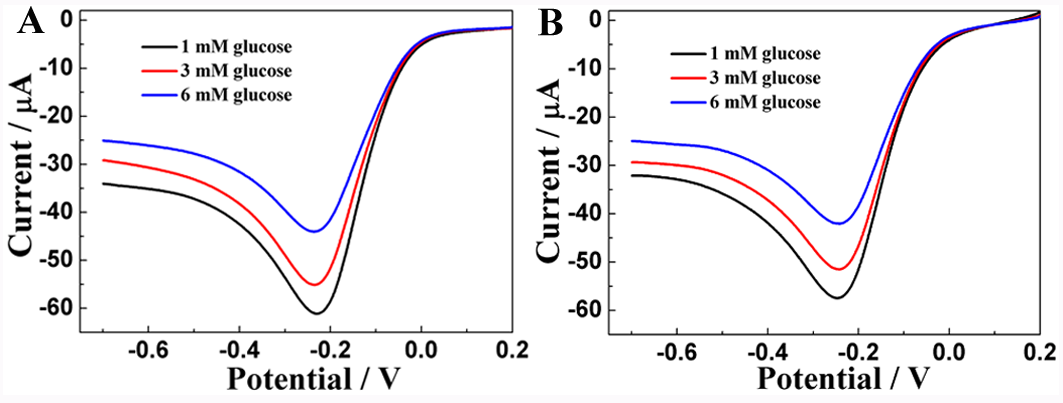


**Figure S5.** LSV of Cu-hemin MOF/GCE in 0.1 M O_2_-saturated PBS (pH=7.0) with (A) free GOD and (B) GOD/Cu-hemin MOF nanocomposite in solution for the catalysis of glucose. Scan rate: 50 mV s^-1^


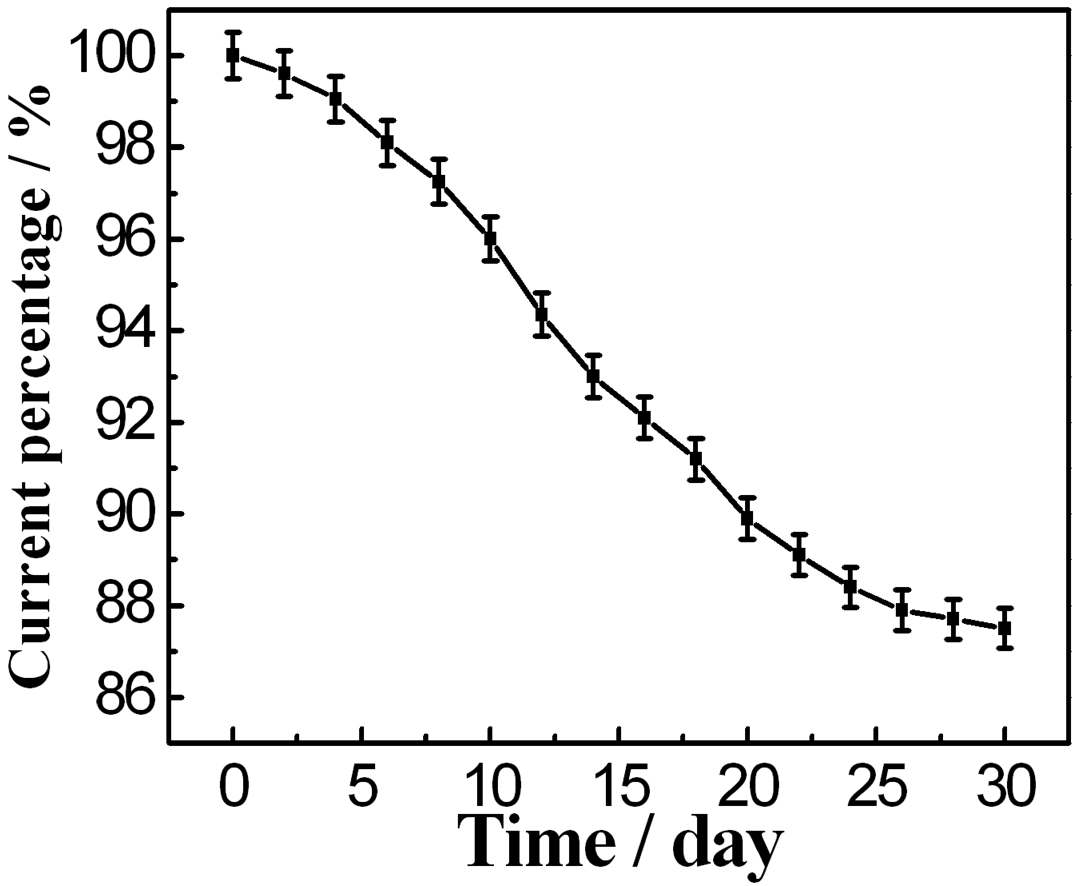


**Figure S6.** The peak current response of GOD/Cu-hemin MOFs/GCE electrode in 0.1 M PBS (pH 7.0) for 30 days.


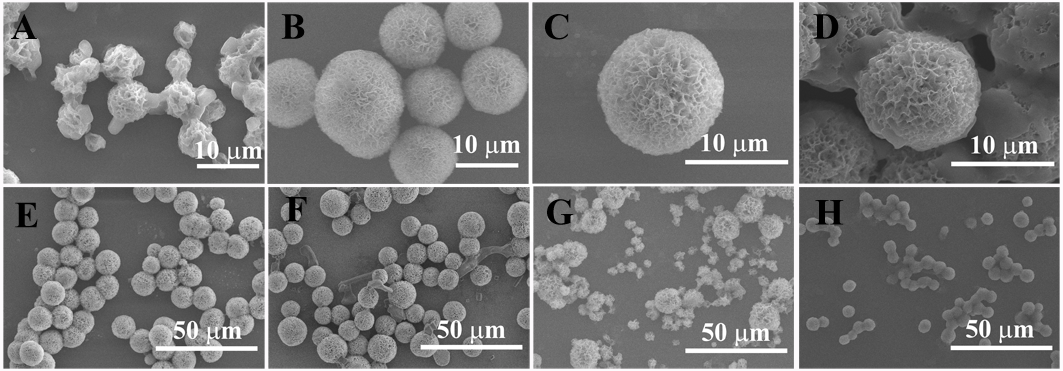


**Figure S7**. (A−D) SEM images of Cu-hemin MOFs with different ratio of Cu^2+^ and hemin (n_Cu2+_ : n_hemin_): 10:1 (A), 108:1 (B), 171:1 (C) and 513 :1 (D). (E−H) SEM images of GOD/Cu-hemin MOFs with different concentration of GOD: 0.5 nmol (E), 1.0 nmol (F), 2.0 nmol (G) and 5.0 nmol (H).

**Table S1. Comparison of the performance of the GOD/Cu-hemin MOFs nanocomposites/GCE with other GOD-based glucose sensors.**

| *Modified electrode* | *Linear range*  *mmol L^−1^* | *Detection limit*  *μmol L^−1^* | *Sensitivity*  *μAmM^-1^cm^-2^* | *References* |
| --- | --- | --- | --- | --- |
| GOD-Cyt c/AuNPs-CHIN/GCE | 2.0-12.0 | 370 | 11.88 | 1 |
| hemin/WS_2_-NSs/GOD/GCE | 0.005-0.2 | 1.5 | −− | 2 |
| AuNPs-GOD/GCE | 2.0-18.0 | 25 | 15.97 | 3 |
| GOx-GNDs/GC | 0.00-0.64 | 1.07±0.03 | −− | 4 |
| PorPC/GOD/GCE | 0.5-18.0 | 80 | −− | 5 |
| GOD-Au-MWCNTs-sG/GCE | 0.05-20.0 | 2.48 | −− | 6 |
| Ag-GOD/GCE | 0.014-3.0 | −− | 13.80 | 7 |
| CS-GOD=ERGO/GCE | 0.02-3.2 | 1.7 | 6.82 | 8 |
| AgNPs-G/GOD/GCE | 2.0-10.0 | 100 | −− | 9 |
| GOD/Cu-hemin MOFs/GCE | 0.009-36.0 | 2.73 | 22.77 | This work |

**Table S2.** Detection of glucose in human serum samples by the GOD/Cu-hemin MOFs nanocomposites/GCE.

| Samples | Samples found  (mM) | Added (mM) | Total Found (mM) | Recovery (%) | RSD (%, n=3) |
| --- | --- | --- | --- | --- | --- |
| 1 | 2.69 | 1 | 3.93 | 106.50 | 2.29 |
| 2 |  | 3 | 5.52 | 97.01 | 4.62 |
| 3 |  | 6 | 8.70 | 100.12 | 3.62 |
| 4 |  | 14 | 16.36 | 98.02 | 3.96 |

**Table S3**. Detection of glucose in human serum samples of diabetes mellitus patients by the GOD/Cu-hemin MOFs nanocomposites/GCE.

| **Sample** | **Found by**  **resulted electrode**  **(mM)** | **Added (mM)** | **Total found (mM)** | **Recovery (%)** | **RSD (%, n=5)** |
| --- | --- | --- | --- | --- | --- |
| **1** | 8.349± 0.003 | 0.5 | 8.852± 0.002 | 100.03 | 3.87 |
| **2** | 8.312± 0.002 | 0.5 | 8.791± 0.003 | 99.76 | 4.58 |
| **3** | 8.301± 0.002 | 0.5 | 8.823± 0.001 | 100.24 | 4.86 |

**References**

(1) Song, Y. H.; Liu, H. Y.; Wang, Y.; Wang, L. *Anal. Methods* **2013,** *5*, 4165-4171.

(2) Chen, Q.; Chen, J.; Gao, C.J.; Zhang, M. L.; Chen, J. Y.; Qiu, H. D. *Analyst* **2015,** *140*, 2857-2863.

(3) Song, Y. H.; Chen, J. Y.; Liu, H. Y.; Song, Y. G.; Xu, F. G.; Tan, H. L.; Wang, L. *Electrochim.Acta* **2015,** *158*, 56-63.

(4) Zhao, M.; Gao, Y.; Sun, J. Y.; Gao, F. *Anal. Chem.* **2015,** *87*, 2615-2622.

(5) Ling, P. H.; Hao, Q.; Lei, J. P.; Ju, H. X. *J. Mater. Chem. B* **2015,** 3, 1335-1341.

(6) Nayak, P.; Santhosh, P.; Ramaprabhu, S. *RSC Adv.* **2014,** *4*, 41670-41677.

(7) Sun, Q. Q.; Yu, Y. N.; Li, J. J.; Bao, S. J. *RSC Adv.* **2015,** *5*, 34486-34490.

(8) Cui, M.; Xu, B.; Hu, C. G.; Shao, H. B.; Qu, L. T. *Electrochim. Acta* **2013,** *98*, 48-53.

(9) Zhang, Y. W.; Liu, S.; Wang, L.; Qin, X. Y.; Tian, J. Q.; Lu, W. B.; Chang, G. H.; Sun, X. P., *RSC Adv.* **2012,** *2*, 538-545.

1. ^^Corresponding author: Tel/Fax: +86 791 88120862/88120861. E-mail: [yhsonggroup@hotmail.com](mailto:yhsonggroup@hotmail.com) (Y. Song) and [lwang@jxnu.edu.cn](mailto:lwang@jxnu.edu.cn) (L. Wang). [↑](#footnote-ref-2)
